# Going, Going, Gone The Diminishing Capacity of Museum Specimen Collections to Address Global Change Research: A Case Study on Urban Reptiles

**DOI:** 10.3390/ani13061078

**Published:** 2023-03-17

**Authors:** Yanlin Li, Anna J. M. Hopkins, Robert A. Davis

**Affiliations:** 1 School of Science, Edith Cowan University, 100 Joondalup Drive, Joondalup, WA 6027, Australia; 2 Department of Terrestrial Zoology, Western Australia Museum, 49 Kew St, Welshpool, WA 6106, Australia

**Keywords:** museum, collections, reptiles, urbanization

## Abstract

**Simple Summary:**

Museums play an important role in research by providing specimens of animals collected across time and space. Traditionally, these collections were used mainly for taxonomy. A contemporary use is to investigate temporal changes in the presence of species during urbanization. We used a study area in Perth, Western Australia, to test whether museum collections of 13 common reptile species were adequate to assess how urbanization in Perth had affected the presence of reptile species. We found that sampling was not adequate to answer this question, with 91% of our study region considered poorly sampled. We encourage a renewed focus on building the collections of museums.

**Abstract:**

It has been increasingly popular to use natural history specimens to examine environmental changes. As the current functionality of museum specimens has extended beyond their traditional taxonomic role, there has been a renewed focus on the completeness of biological collections to provide data for current and future research. We used the collections of the Western Australian Museum to answer questions about the change in occurrence of five common reptile species due to the rapid urbanization of Perth. We recorded a significant decline in collection effort from the year 2000 onwards (F = 7.65, *p* < 0.01) compared to the period 1990–1999. Spatial analysis revealed that only 0.5% of our study region was well sampled, 8.5% were moderately sampled and the majority of the regions (91%) were poorly sampled. By analysing the trend of specimen acquisition from 1950 to 2010, we discovered a significant inconsistency in specimen sampling effort for 13 common reptile species across time and space. A large proportion of past specimens lacked information including the place and time of collection. An increase in investment to museums and an increase in geographically and temporally systematic collecting is advocated to ensure that collections can answer questions about environmental change.

## 1. Introduction

The field of natural sciences rapidly expanded in the 19th century [1]. As a result, efforts in the collection and preservation of natural history specimens increased exponentially [2,3], resulting in large-scale specimen collections which provide a basis for current and future research on all aspects of biological and environmental science. These specimens are primarily used for taxonomic and systematic studies [4] and they provide a basic reference point for taxonomists, allowing for the reinterpretation of existing species and phenotypes [5]. Individual specimens contain unique information, documenting the biology and physiology of the organism, as well as the environmental information from the time and location at which that individual was collected [6,7]. They also document the connection that individual specimens had with other specimens collected from the same period. These properties of specimens make them important resources for studying the dynamic interactions and monitoring the fluctuations within ecological communities [8,9]. When well-prepared, a specimen can remain a scientific asset for hundreds of years [10].

In the Anthropocene, humans are held responsible for the changes in Earth’s biochemical process, decrease in biodiversity and the alteration in ecological interactions [11]. Research associated with global change biology has been receiving growing attention; however, it is challenging to quantify the impacts humans have had on the environment due to the dearth of effective indicators to track human-driven changes [12]. Historically, the primary source of data for global change biology was obtained from field expeditions; however, such data were limited to a narrow range of localities, time frames and taxa [13]. Due to a general decline in the collection of specimens in contemporary times [14,15] and a desire to look at changes over time, it has been increasingly popular to use natural history specimens to examine environmental changes [16,17,18].

As the current functionality of museum specimens has extended beyond their traditional taxonomic role, they have become prominent indicators for environmental change with the aid of modern technologies [12]. For example, the spread of harmful pathogens or diseases due to anthropogenic activities can be tracked using museum specimens [19,20]; indeed, the origin and spread of the chytrid fungus *Batrachochytrium dendrobatidis*, which is responsible for the drastic decline of global anuran populations, has been successfully tracked using natural history specimens [21,22]. Long-term collection series can also track the infiltration of contaminants into the environment [23,24]. Furthermore, natural history specimens can also help track the fluctuation in species diversity and abundance across a broad scale of time and space [18,25], including the arrival and colonization of vagrant species due to climate shifts [26].

Urbanization research has become a key component of global change biology. There has been a renewed focus on how urbanization has impacted wildlife and how wildlife has responded to it behaviourally, biologically and physiologically. The most common method used to investigate these questions is to compare an urban population to its rural counterparts [27,28,29]. While these spatial analyses provide critical insight into how populations differ, they do not give us details of the drivers of change. In contrast, temporal studies can provide an understanding of how different selective pressures have acted on organisms and can highlight the evolutionary processes [30]. By comparing historical and contemporary specimens, studies have demonstrated how urbanization can affect behaviour [31,32], morphology [33,34] and life cycles [35]. The effectiveness of such studies is dependent on the consistency of specimen collection over space and time. Unfortunately, a decline in the acquisition of natural history specimens has been detected among museums globally [14,15].

As a case study, to investigate the utility of museum collections as a resource for examining change, we compare specimen collection patterns between urban and rural areas within the study region of urban Perth, Western Australia. Specifically, we focus on the most common reptile species in the region and interrogate the data to see if collections have the sampling resolution required to detect changes due to rapid urbanization in the region.

## 2. Materials and Methods

### 2.1. Specimen Source

Data were drawn from the herpetology collection of the Collection and Research Center of Western Australian Museum. Although this collection includes multiple sources of specimen acquisition, by far the majority of specimens are from dedicated fauna surveys with targeted vouchering or unexpected trap deaths which have then been lodged with the museum. Such collecting was carried out under various ethics and government wildlife department approvals. The predominant method of specimen preservation is wet specimens (fixed in formalin and then preserved in ethanol). This method maintains the flexibility of the specimens, which distinguishes herpetofauna specimens from most bird, mammal and insect specimens. One advantage is that this preservation method allows for an easy examination of the specimen’s external and internal traits. The Collection and Research Center of the Western Australian Museum holds the largest wet specimen collection in the southern hemisphere.

### 2.2. Study Species and Location

**Figure 1:**
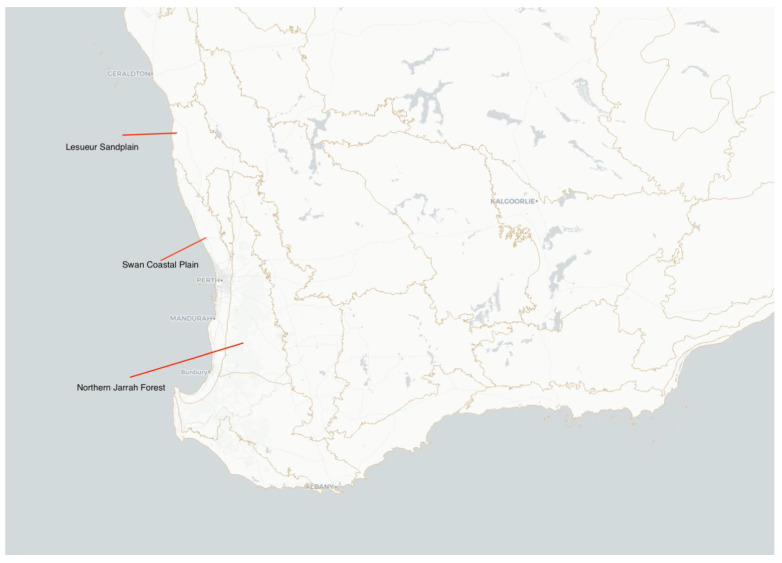
Map of the Swan Coastal Plain showing the Perth metropolitan region (light grey, labelled Perth) and the adjacent bioregions. Source: nationalmap.gov.au, accessed on 3 February 2023.

The Perth metropolitan region of Western Australia is situated on the Swan Coastal Plain (SCP), bounded by the Indian Ocean on the west, and the Darling Range to the east (Figure 1). There are 54 species of lizards inhabiting Perth and adjacent bioregions [36]. Previous work on reptiles in this region is well documented in [37] and includes recent range extensions and taxonomic work on reptiles. For our study, we selected species whose historical distribution overlapped with the current extent of the Perth metropolitan region. Species with few records from the SCP were excluded. Next, we expanded our scope to include bioregions adjacent to the SCP, but outside the metropolitan extent, namely, the Lesueur Sand Plain (sub-bioregion of Geraldton Sand Plain) and Northern Jarrah Forest (sub-bioregion of Jarrah Forest; Figure 1). Desirable candidate species needed to have a natural distribution throughout these regions and would provide an adequate amount of specimens in both the current metropolitan extent and adjacent non-metropolitan areas. We ranked specimen collections based on the most frequently represented species and used these for further analyses.

### 2.3. Temporal and Spatial Analyses

The data were refined by excluding specimens that lack a collection date or GPS coordinates. As specimens can sometimes take as long as a decade to be catalogued into the museum’s database, we limited our study to specimens collected before 2010. For temporal analyses, we used the time of collection for each specimen to rank specimens obtained each year between 1950 and 2010, in order to examine the trend of specimen collection through time. To gain a better understanding of long-term collection trends, we calculated a moving average based on a 5-year interval because annual fluctuations can be difficult to interpret, and the 5-year moving average allowed for a more comprehensive exploration of the specimen acquisition across time. Next, we used a quadratic polynomial regression from the 5-year moving average (excluding 2011 until the present year) to summarize the overall specimen acquisition trend between 1950 and 2010. Finally, we calculated the percentage of increase/decrease in specimens acquired in a 5-year period compared to the previous 5-year period. Because the majority of specimens collected in the period 1950 to 1954 lack georeferencing, if the small number of georeferenced records were included, preliminary analysis showed that the next period (1955~1959) would have a 1380% increase in specimen acquisition. To avoid skewing the result, the period 1950 to 1954 was excluded from this analysis.

For spatial analyses, all records with GPS coordinates were used regardless of the year of collection, and an approximate georeferenced record was deemed acceptable. We created a map layer of the selected bioregions on ArcGIS Pro version 2.9, and divided it into equally sized quadrats using the Fishnet function [38]. The outer-rim of the quadrats overlapped with the borders of the bioregions in order to include all the individuals near the edges. We tallied the total number of georeferenced specimens and sorted them into the gridded bioregional map. The density of specimens within each quadrat was calculated, then depicted across 6 bins using a color-coded system, ranging from absent (sample size = 0) to high (sample size > 100). This allowed for us to explore the variation in the spatial coverage of specimens collected. Finally, we explored the spatio-temporal trends of specimen acquisition by examining specimen distribution and density across six 10-year periods, 1950~1959, 1960~1969, 1979~1979, 1980~1989, 1990~1999, 2000~2010 (the last period contains 11 years).

### 2.4. Statistical Analyses

We used Analysis of Variance (ANOVA) to examine the temporal patterns of specimen collection for all selected species. To evaluate the differences between decades, we used Tukey’s post hoc test (HSD). A Wilcoxon test was used to determine whether the decadal mean significantly deviated from the overall mean (overall mean = average annual sample collected between 1950 and 2010). Lastly, we used a chi-squared test to examine whether the specimen proportion deviated from the taxonomic diversity of the selected species.

## 3. Results

After applying the filters, there were 13 species that matched our criteria, and this equated to more than 16,000 specimen records. After removing the records that lack the collection date and georeferenced location, we were left with 14,907 records, regardless of the year of collection. Of those specimens, 6279 records were collected within the selected bioregion complex.

**Figure 2:**
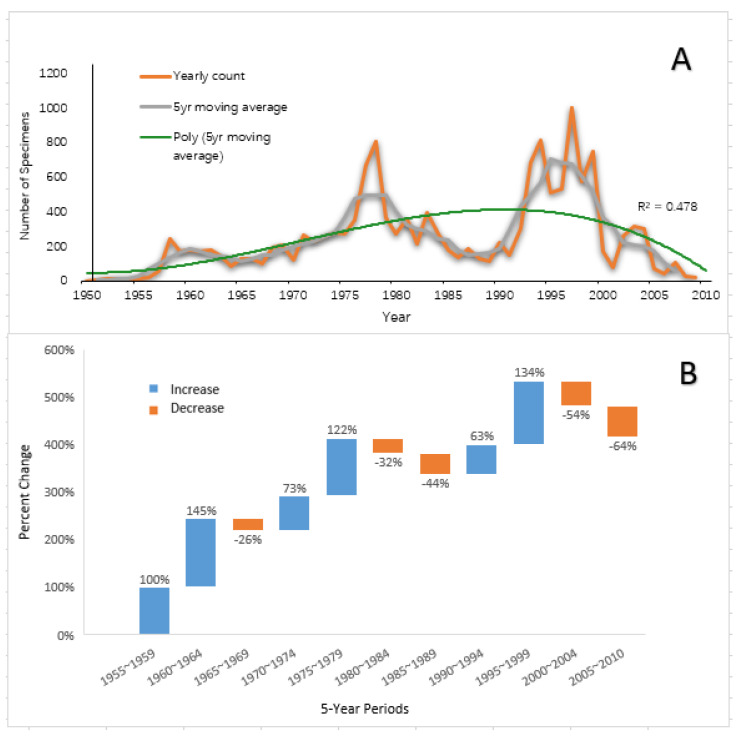
(**A**) Temporal trends of specimen acquisition for the 13 study species in the study region; the orange line represents the annual count of specimens collected, with the grey line representing the 5-year moving average. The green line represents a quadratic polynomial that is a best fit for the 5-year moving average. (**B**) Percentage changes in trends of the specimen acquired per 5-year period compared to the previous period.

The data indicated that large-scale specimen collection and preservation for the 13 lizard species appear to have been initiated in the 1950s, the aggregated growth of specimen collection displayed an increasing trend for more than 30 years and then the upward trend began to level out in the mid-1990s before decreasing drastically by the end of the 20th century (Figure 2). By the end of the investigation period (2010), annual specimen acquisition was equivalent to a pre-1960s level (Figure 2A).

A Wilcoxon signed-rank test revealed that the decline in collection observed from the year 2000 onwards was significant (F = 7.65, *p* < 0.01) compared to the previous decade (1990~1999). Between 2000 and 2010, 2122 specimens were added to the collection, a number comparable to a pre-1970 level. Overall, the specimen collection rate peaked during the late 1970s and 1990s, before falling to an exceptionally low rate in recent decades (Figure 2B).

The overall mean was approximately 242 specimens collected per year. Results from the Wilcoxon test indicate that the decadal means of the 1950s and 1960s were significantly lower than that of the overall mean (mean = 36 and 145, respectively), while the decadal means of the 1970s and 1990s were significantly higher than that of the overall mean (mean = 344 and 390, respectively). The decadal mean of 1980s and 2000s were 251 and 193, respectively, and they did not differ significantly from the overall mean.

**Figure 3:**
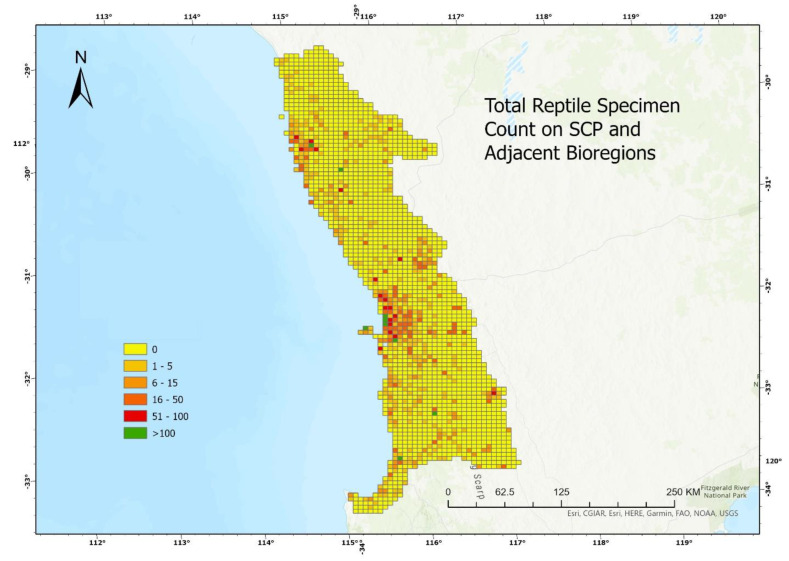
Total specimen acquisition for the 13 study species plotted against the bioregion complex map to show the pattern of spatial variation, using 6 coloration levels from 0 specimens collected within the quadrat to more than 100 specimens collected within the quadrat.

Using the ‘Fishnet’ function from the software ArcGIS Pro, the bioregion complex was divided into 2117 equally sized quadrats; spatial mapping for all of the specimens of the 13 species, revealed an uneven sampling pattern across the entire study region. Quadrats containing 1 to 10 specimens were considered poorly sampled regions, quadrats containing 11 to 50 specimens were considered moderately sampled regions and quadrats containing more than 50 specimens were considered well-sampled regions. Among the 2117 quadrats, only 0.5% were well-sampled regions, 8.5% were moderately sampled and the majority of the regions (91%) were poorly sampled. A large number of specimens were concentrated around the Perth metropolitan region and the southern limit of the Lesueur Sand Plain, where it meets the border of the Swan Coastal Plain (Figure 3).

**Figure 4:**
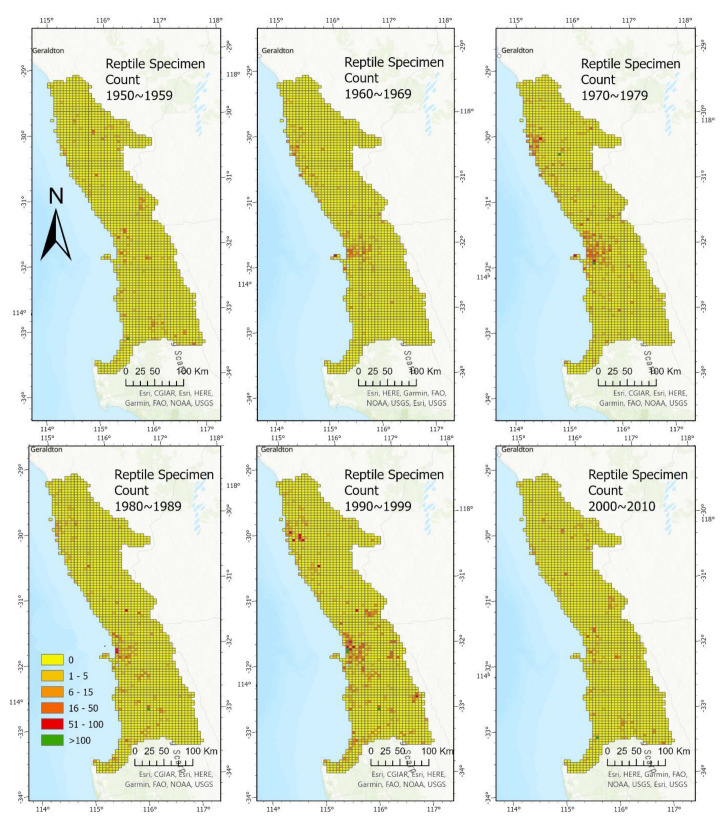
Specimens of the 13 study species acquired per decade (1950–1959; 1960–1969; 1970–1979; 1980–1989; 1990–1999; 2000–2010), plotted against the bioregion complex map in a descending chronological order to show the spatio-temporal trends of specimen collection over these time periods. Each individual map used the usual 6 colouration levels from zero specimens collected within the quadrat to more than 100 specimens collected within the quadrat.

Despite the comparatively high sampling effort in the 1970s and 1990s, the spatiotemporal map showed that none of the decades have sufficiently sampled the entire bioregion complex. The Perth metropolitan region has been sampled consistently until the end of the 20th century; however, specimens are lacking from recent decades (Figure 4).

**Figure 5:**
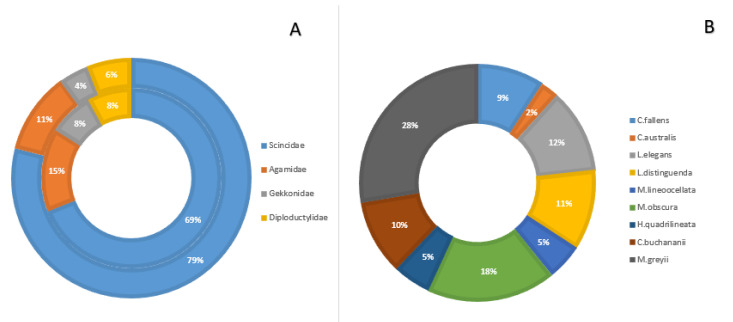
(**A**) Proportional comparison—the outer ring represents the percentage of the total amount of specimens per lizard family, and the inner ring represents the percentage of the diversity of each family. (**B**) Specimen percentage of the 9 lizard species of the family Scincidae.

Among the thirteen target species in this study, nine species (69%) were from the family Scincidae, two (15%) belong to the family Agamidae, and the family Gekkonidae and Diplodactylidae were represented by one species (8% of total) each (Table 1). We expected a similar proportion for the total amount of specimens collected, and the chi-squared test result showed that this proportion did not deviate significantly from the species diversity (*p* = 0.17, Figure 5A). However, an uneven proportion of specimens was observed within the Scincidae family, but a chi-squared test could not be performed because the expected proportion cannot be calculated as the abundance of different species might vary naturally (Figure 5B).

## 4. Discussion

Natural history specimens will continue to play a traditional role as the foundation for classifying new species and clarifying phylogenetic relationships. Australia is a world hotspot for reptile diversity, especially in the arid regions [39,40], where native species have shown a high-level of fine-scale taxonomic divergence [41]. Consequently, new species continue to be described by analysing existing museum specimens and those recently collected from poorly sampled regions [42,43,44,45,46,47,48]. As demonstrated by the current study, the advent of new research fields, such as the impact of urbanization on animals, places an increase in demand for these specimens; however, the collections may not always be adequate to answer these questions. There is a need to reflect on the current trend of the collection and curation process.

By analysing the trend of specimen acquisition from 1950 to 2010, we discovered a significant inconsistency in the specimen sampling effort for the 13 common study species across time and space. A large proportion of the specimens prior to the 1960s lack general information including the place and time of collection, but very few specimens lack this information from 1960 onwards. Other studies have reported similar findings. A study using VertNet, which contains information on approximately 1.6 million mammal specimens (excluding digital records and fossils) from museums all over United States, revealed that at least 6% of specimens are without a date of collection and an astonishing 34% lack geo-referencing [14]. In comparison, in our study, these data were generally not lacking and were not a barrier to the use of specimen data.

Spatially, more than 90% of our ex-urban study area was poorly sampled, with zero or less than ten specimens. In contrast, the Perth metropolitan region had a comparatively high specimen concentration. This is likely because the metropolitan region is near to scientific institutes such as universities, conservation organizations and museums, and has more infrastructure developments requiring environmental impact assessment surveys, and therefore, more biological surveys took place. It is common for areas that are remote and have low accessibility to receive less sampling effort [14]. However, compared to other remote regions in Western Australia, there are a reasonable number of specimens collected through field studies in the southern portion the of Lesueur Sand Plain, which demonstrates that when strategically planned, sample collection from remote regions is achievable.

Temporally, though suffering a marked decrease in the 1980s, specimen acquisition rates peaked between the 1970s and 1990s, before steeply declining by 118% since the start of the 21st century. This result partially compares with a similar study. A meta-analysis of 110 years of specimen deposition trends of terrestrial vertebrates in the United States found that apart from birds, of which the decrease in specimen acquisition was less pronounced, a sizeable decline was observed for amphibians, reptiles and mammal after an acquisition rate peak in the late 1960s [15]. Results from the current study show that the aggregated growth of specimen acquisition for the 13 species lasted until the end of the 1990s.

Our study collection had an equal balance in the proportion of specimens between the four lizard families considered in this study; however, the proportion was unbalanced within the Scincidae. For example, *Ctenotus fallens* represents 9% of the specimens while *C. australis* was only represented by 2%. Historically, these two species were equal in abundance at many reserves around Perth [49]. However, a more recent survey showed that the population of *C. fallens* remained stable, while *C. australis* was nearly absent in some of the parks and reserves [50,51]. Therefore, the sampling disparity between closely related species could be a reflection of actual decline in species abundance. However, sampling techniques or targeted species sampling will influence representation within a collection and caution is needed when drawing inferences on abundance.

Overall, in our study, it is evident that the specimen collection for the 13 species considered is in steep decline, and this is caused by a series of complex and inter-related factors. It is a common sentiment that small and vulnerable populations should not be heavily sampled to avoid population declines due to over-collecting. Studies on both long- and short-term collections of small mammals do not all support the perception that specimen collection will negatively impact on populations [52,53]. Furthermore, it has been shown that reptile populations are usually resilient to responsible regular sampling [54] and a responsible and strategically planned sample collection should not reach the threshold of endangering natural populations. The number of specimens collected annually is dwarfed by the number of animals lost each year due to road collisions, predation by invasive species and land clearing [55,56,57]. One study estimated densities of reptiles in the kwongan heathlands of Western Australia of up to 958.3 individuals/ha, indicating potentially high densities, large populations and a very high rate of mortality due to land clearing for any developments [58]. Many Australian reptiles are threatened due to their naturally narrow distribution, and therefore, are present in smaller populations [59]. Specimen collection should be strategically planned, with clear goals, to minimise disturbance to populations.

Aside from these constraints, limitations on the regular collection of common species could severely impede future research, management and conservation plans since whole specimens are key to a number of research questions [60]. Any advocacy for increased specimen collecting will require responsible departments reviewing regulation and permitting protocols, maintaining the current level of restriction on endangered species, while loosening the restriction on common species. A further problem arises in that since strict requirements for permits and ethics are required for collecting specimens, it precludes the role of the general public, apart from those species large enough to be subject to roadkill and thus collected by the public. In our example, this would include only two species—the venomous brown snake, the dugite *Pseudonaja affinis* [61], and the varanid lizard, *Varanus gouldii*. Most of the species in our dataset are small, restricted to bushland areas and have small home ranges. The outcome of this is that museums and research institutions will continue to be the primary drivers in specimen collections in the future.

Taxonomy is an important consideration in sampling design and execution, of which there is a need to account for the dynamic nature of species identity. A number of species represented in our study region have been split as cryptic species complexes, e.g., *Crytoblepharus* [62], and a new species *Ctenotus ora* was described from the SCP due to genetic analyses [63]. An increase in taxonomic effort is required to redocument existing species in collections based on these changes and to incorporate such knowledge into the design of sampling programs. Most museums also face a backlog of specimens for accession and documentation as well as taxonomic changes. It is essential that museums are funded to enable sufficient resources to address these shortfalls given the recognized current and looming crisis in taxonomy of a lack of skilled staff and insufficient funding [64,65]. The advent of new and improved methods for detecting cryptic species, combined with the knowledge that there are certainly many undocumented species yet to be described [66], all emphasize the critical role of taxonomy in museum collecting.

Although non-lethal or less invasive, data-gathering techniques could produce data on species’ presence [67]; these forms of data such as photographs, videos and even tissue samples, do not capture data such as external morphology, skeletal structure or internal anatomy—all of which may be required for taxonomic studies. Some studies have advocated that observational data often fall short of providing integrated data in order to yield critical information of an animal [68]. Nonetheless, a large-scale sample collection is not always possible because some species are endangered or have naturally small populations, so citizen science and observational data may still have an important role in lieu of actual specimens.

## 5. Conclusions

Finally, in planning for the future, a major drive for the decline in the acquisition of specimens after the 20th century is the lack of financial investment in museum field work. Research involving organisms often depend on the infrastructure curated at the museum. For instance, in the *Journal of Mammalogy* in the USA, a least 25% of their articles directly benefited from museum collections [69]. If we wish to continuously benefit from museums, it is essential that budgets should be increased. Existing biologists in private and public industries often undertake fauna surveys as part of environmental impact assessments [70]; they should work closely with museums, consider regular sampling for each project to increase collections and address both spatial and temporal gaps. The role of taxonomists is more important than ever, and they should have a key role in planning sampling programs to test hypotheses on speciation and diversity.

## Figures and Tables

**Table 1:** List of reptile species on the Swan Coastal Plain with enough records for further analysis.

Species	Family
*Christinus marmoratus*	Gekkonidae
*Cryptoblepharus buchanani*	Scincidae
*Ctenophorus adelaidensis*	Agamidae
*Ctenotus australis*	Scincidae
*Ctenotus fallens*	Scincidae
*Hemiergis quadrilinieata*	Scincidae
*Lerista distinguenda*	Scincidae
*Lerista elegans*	Scincidae
*Menetia greyii*	Scincidae
*Morethia lineocellata*	Scincidae
*Morethia obscura*	Scincidae
*Pogona minor*	Agamidae
*Strophurus spinigerus*	Diplodactylidae

## Data Availability

Data analysed are existing data from the WA Museum and are available on request to the WA Museum.
